# Novel Roles for Selected Genes in Meiotic DNA Processing

**DOI:** 10.1371/journal.pgen.0030222

**Published:** 2007-12-07

**Authors:** Philip W Jordan, Franz Klein, David R. F Leach

**Affiliations:** 1 Institute of Cell Biology, University of Edinburgh, Edinburgh, United Kingdom; 2 Department of Chromosome Biology, University of Vienna, Vienna, Austria; University of North Carolina at Chapel Hill, United States of America

## Abstract

High-throughput studies of the 6,200 genes of Saccharomyces cerevisiae have provided valuable data resources. However, these resources require a return to experimental analysis to test predictions. An in-silico screen, mining existing interaction, expression, localization, and phenotype datasets was developed with the aim of selecting minimally characterized genes involved in meiotic DNA processing. Based on our selection procedure, 81 deletion mutants were constructed and tested for phenotypic abnormalities. Eleven (13.6%) genes were identified to have novel roles in meiotic DNA processes including DNA replication, recombination, and chromosome segregation. In particular, this analysis showed that Def1, a protein that facilitates ubiquitination of RNA polymerase II as a response to DNA damage, is required for efficient synapsis between homologues and normal levels of crossover recombination during meiosis. These characteristics are shared by a group of proteins required for Zip1 loading (ZMM proteins). Additionally, Soh1/Med31, a subunit of the RNA pol II mediator complex, Bre5, a ubiquitin protease cofactor and an uncharacterized protein, Rmr1/Ygl250w, are required for normal levels of gene conversion events during meiosis. We show how existing datasets may be used to define gene sets enriched for specific roles and how these can be evaluated by experimental analysis.

## Author Summary

Since the genome of S. cerevisiae was sequenced in 1996, a major objective has been to characterize its 6,200 genes. Important contributions to this have been made using high-throughput screens. These have provided a vast quantity of information, but many genes remain minimally characterized, and the high-throughput data are necessarily superficial and not always reliable. We aimed to bridge the gap between the high-throughput data and detailed experimental analysis. Specifically, we have developed a strategy of combining different sources of high-throughput data to predict minimally characterized genes that might be implicated in DNA processing. From this we have gone on to test the involvement of these genes in meiosis using detailed experimental analysis. In a sense, we have turned high-throughput analysis on its head and used it to return to low-throughput experimental analysis. Using this strategy we have obtained evidence that 16 out of 81 genes selected (20%) are indeed involved in DNA processing and 13 of these genes (16%) are involved in meiotic DNA processing. Our selection strategy demonstrates that different sources of high-throughput data can successfully be combined to predict gene function. Thus, we have used detailed experimental analysis to validate the predictions of high-throughput analysis.

## Introduction

Meiotic DNA processing includes molecular functions such as DNA replication, repair, recombination, chromosome modification, and segregation. The fidelity of DNA processing events during meiosis is critically important as errors can give rise to mutations, genome rearrangements, and aneuploidies that are associated with genetic disorders.

A large number of high-throughput analyses have been performed to characterize the 6,200 genes of S. cerevisiae. These have included genomic screens for protein–protein [1–3] and protein complex interactions [4–7], high-throughput genetic interaction analyses [8–13], genome-wide measurements of gene expression under various environmental conditions [14–19], comprehensive measurements of subcellular localization of proteins [20,21], and assessments of deletion phenotypes of single genes [22–24]. Although these high-throughput datasets have proved to be useful, at the time of this work more than one third of the S. cerevisiae genes did not have a biological process and/or molecular function assigned on the *Saccharomyces* Genome Database (SGD) [25]. One major drawback of high-throughput studies is the difficulty in assessing the large amount of data that are produced, and to compound the problem further, spurious data are common [26,27]. However, it has been shown that problems with false information within datasets can be circumvented by combining data from different high-throughput experiments, as the data can either support or contradict one another [28,29].

In this report, a strategy of combining high-throughput data available for protein and genetic interactions, protein subcellular localization, and mRNA expression patterns, together with data from phenotype experiments, was used to identify minimally characterized genes potentially implicated in DNA processing. Homozygous deletion mutants were made for 81 genes selected with the data integration strategy and were assessed to detect roles in meiotic DNA processing. As a result, eleven (13.6%) genes were found to have novel roles in meiotic DNA processing.

## Results

### Integration of Datasets to Select Genes with Roles in DNA Processing

**Figure 1:**
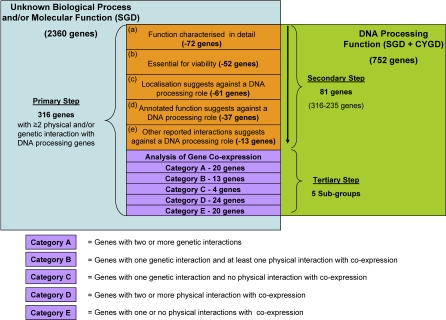
Integration of Datasets to Select Genes with Roles in DNA Processing See text and Figure S1 and Tables S1, S2, and S3 for details of the selection strategy.

An in-silico selection strategy (Figure 1) was designed to combine high-throughput datasets, to identify mutants conferring DNA processing phenotypes. 81 genes (3.4% of the minimally characterized genes in the genome) were selected for further analysis. During primary selection, genes not annotated for a biological process and/or molecular function (minimally characterized genes) were selected if either a genetic or physical interaction partner involved in DNA processing could be identified. A gene was defined to be involved in DNA processing if its annotation was related to one or more of the following functions: DNA replication, repair, recombination, and related checkpoints, as well as chromosome segregation and chromatin structure/modification by the Comprehensive Yeast Genome Database (CYGD) or the Saccharomyces Genome Database (SGD). In this way a list of 752 DNA processing genes was created (Table S1). To increase stringency we required a minimum of two DNA processing interaction partners, which reduced the number of candidates from 718 to 316 genes. The interaction data were taken from the Yeast General Repository for Interaction Datasets (GRID) [30] and Database of Interacting Proteins (DIP) [31].

The secondary selection aimed to select against genes that had unfavourable characteristics. Of the initially chosen genes, 72 had well documented roles in DNA processing and were therefore removed (e.g., *MAD1*, well characterized for its role in the spindle checkpoint [32] and *ZIP2*, which has been shown to be an intrinsic component of the synaptonemal complex [33]). Of the genes essential for vegetative growth, 52 were not assessed. A further 61 genes were removed because of protein localization inconsistent with roles in DNA processing (e.g., mitochondria, endoplasmic reticulum, cell wall, bud neck, endosome, Golgi apparatus, vacuole, or lipid).

Also excluded were 37 genes annotated for roles in cell wall organization and biogenesis, bud site selection, vacuole transport, and nutrient metabolism. We excluded 13 genes because the fraction of their interaction partners involved in DNA processing was less than 1/5. The secondary selection resulted in the identification of 81 genes that were all subsequently analyzed experimentally (see Table S2 for the list of genes removed during the secondary selection).

It has been reported that genetic interaction data have a much higher confidence than physical interaction data [34], and it has been observed that mRNA expression patterns often correlate for proteins that interact physically [35–38]. To assess the 81 candidates further, the gene expression correlations of all physical interaction pairs were compiled. The method of assessing mRNA correlation used here has been described and assessed previously [29]. This method calculates a cosine correlation distance for a pair of proteins that is between zero for complete correlation, and two for anti-correlation. A correlation distance of below 0.9 was deemed sufficient expression correlation to support the interaction. This cut-off was decided for two reasons; firstly, yeast two-hybrid data generally have a weak relationship with gene expression correlation [38] and therefore a cut-off value too stringent would miss true interactions. Secondly, in general a correlation distance over 0.9 did not successfully predict interactions [29]. Using this information, the 81 selected genes were subdivided into five categories (Figures 1 and S1; Table S3 for all data and examples of this selection step). Category A consisted of 20 genes that possessed two or more genetic interactions with DNA processing genes. The 13 genes of Category B had a single genetic interaction and at least one physical interaction that showed correlated expression. The four genes of Category C had a single genetic interaction and at least one physical interaction without correlated expression. Category D consisted of 24 genes that had two or more physical interactions with correlated expression, and the remaining 20 genes of Category E had at least two physical interactions that do not have correlated expression.

### Overview of the Screen

Deletion mutants for the 81 genes arising from our secondary selection were created in *MAT*-a and *MAT*-alpha W303 backgrounds. The experimental screen included testing for sensitivity to hydroxyurea (HU), methyl methanesulphonate (MMS), and X rays during vegetative growth, as well as assessing sporulation efficiency, meiotic nuclear division, spore viability, and levels of meiotic chromosome missegregation and gene conversion (Figure S3 and Table 1).

**Table 1:**
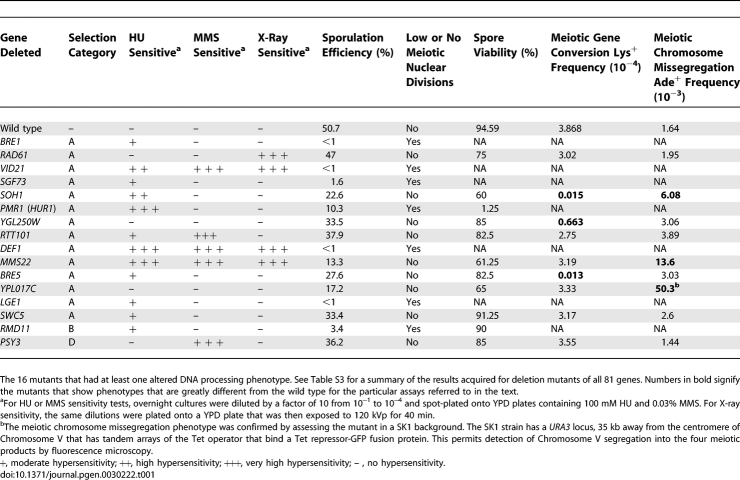
Summary of the Deletion Mutants for 16 of 81 Selected Genes That Had an Altered DNA Processing Phenotype

Twelve deletion mutants were shown to have increased sensitivity to HU, four of which were also sensitive to MMS, and three to X rays (Table 1). Additionally, two mutants, *rad61Δ* and *psy3Δ*, not sensitive to HU, were sensitive to X rays and MMS, respectively (Table 1). Results presented here are consistent with at least one previous genome-wide screen [39–43], with the exception of three mutants that show mild sensitivity to HU, *sgf73Δ*, *swc5Δ*, and *rmd11Δ*. These phenotypes were shown to be the same in both *MAT*-a and *MAT*-alpha haploid strains.

**Figure 2:**
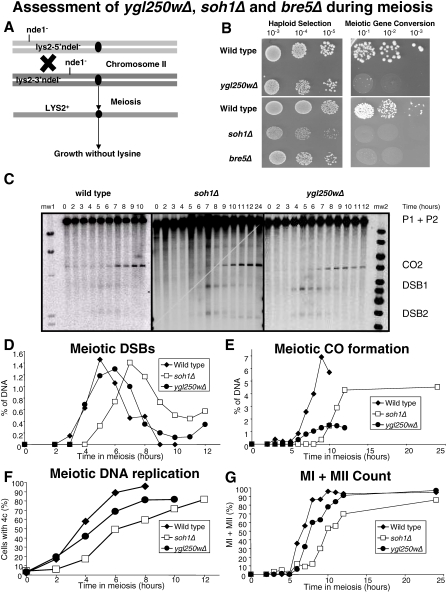
Assessment of *YGL250W*, *SOH1*, and *BRE5* (A) Schematic representation of Chromosome II from the diploid W303 background which consists of two *LYS2* heteroalleles (*lys2–5′nde*I*^−^* and *lys2–3′nde*I*^−^*). These were used to measure meiotic gene conversion (see Materials and Methods). (B) Spot test of wild type, *ygl250wΔ*, *soh1Δ*, and *bre5Δ* on haploid selection plates and haploid selection plates without lysine to measure meiotic gene conversion. The reduction in meiotic gene conversion of *ygl250wΔ*, *soh1Δ*, and *bre5Δ* was further assessed by random spore analysis (Table 1). (C) Southern blot of DNA isolated from wild type, *soh1Δ*, and *ygl250wΔ* SK1 strains containing the ectopic *URA3*-*ARG4* interval on Chromosome III. The DNA from the indicated times after initiation of sporulation were digested with XhoI then probed to detect COs and DSBs; mw1 represents the λ-HindIII molecular weight marker (Fermentas) and mw2 represents the 1-kb molecular weight marker (Fermentas). The full-sized Southern blots are presented in Figure S1. For graphs (D–G), wild type, *ygl250wΔ*, and *soh1Δ* are represented by black diamonds, black circles, and white squares, respectively. The corresponding XhoI-digested Southern blots are presented in Figure 3C. (D) Pre-meiotic DNA replication was assessed for synchronized meiotic cultures by fluorescence-activated cell sorting (FACS) and the change from 2c to 4c DNA content was plotted over time. (E) Nuclear divisions (MI and MII) of the synchronized meiotic cultures in (E) were assessed with fluorescence microscopy using 4′,6-diamidino-2-phenylindole (DAPI) staining to visualize nuclear division. (F) Molecular analysis for DSB (DSB1) signal/total lane signal from Southern blots of DNA extracted from synchronized meiotic cultures. (G) Molecular analysis for CO (CO2) signal/total lane signal from Southern blots of DNA extracted from synchronized meiotic cultures.

Meiotic missegregation of Chromosome 1 was quantified by selecting spores which carry both *ADE1* and *ade1::ADE2* alleles indicating the presence of a second chromosomal copy (see Materials and Methods). Three mutants displayed increased levels of meiotic Chromosome 1 missegregation: *soh1Δ* (5-fold), *mms22Δ* (10-fold), and *ypl017cΔ* (35-fold) (Table 1). Meiotic gene conversion was measured by restoration of a functional *LYS2* gene from *lys2–5′nde*I*^−^* and *lys2–3′nde*I*^−^* heteroalleles (Figure 2A). Spot tests and random spore analysis revealed a reduced level of gene conversion compared to the wild type (Figure 2B, Table 1) for *ygl250wΔ* (6-fold), *soh1Δ* (>250-fold), and *bre5Δ* (>250-fold). Further analysis of these mutants is discussed below. Six mutants (*bre1Δ*, *vid21Δ*, *sgf73Δ*, *rmd11Δ*, *def1Δ*, and *lge1Δ*) were found to have very low or no nuclear divisions in meiosis (Table 1). Further analyses of these genes' roles in DNA processing are discussed below.

**Figure 3:**
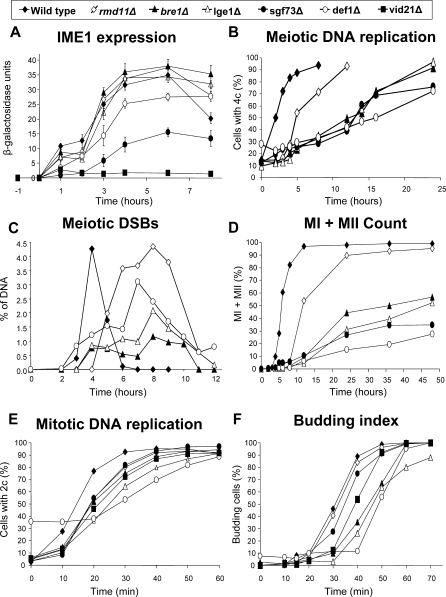
Further Characterization of *VID21*, *BRE1*, *LGE1*, *RMD11*, *SGF73*, and *DEF1* Mutants for these genes were made in an SK1 background. The plots on each graph represent wild type (black diamonds), *rmd11Δ* (white diamonds), *bre1Δ* (black triangles), *lge1Δ* (white triangles), *sgf73Δ* (black circles), *def1Δ* (white circles), and *vid21Δ* (black squares). Where error bars are not shown, the time courses are of individual experiments. A total of three experiments were carried out in each case and the data shown are consistent with those obtained in the other experiments. (A) The expression of *IME1*, a primary transcription factor required for entry into the meiotic cell cycle was assessed. SK1 strains carrying a plasmid that expresses the *lacZ* reporter gene under the control of the *IME1* promoter were grown for synchronous meioses and assessed for *lacZ* expression via β-galactosidase activity [92]. W303 *MAT*-a mutant strains for the above genes were assessed for G1 to S phase transition in mitosis after release from α-factor arrest [87]. (B) Pre-meiotic DNA replication was assessed for synchronized meiotic cultures by FACS and the change from 2c to 4c DNA content was plotted over time. See Figure S2 for the raw data of the FACS analysis for meiotic DNA replication. (C) DNA extractions from sporulation time courses were digested with BglII and meiotic DSB formation (DSBIII and IV) at the *THR4* hotspot was assessed using Southern blotting and probing techniques [46]. See Figure S3 for the *THR4* Southern blots. (D) Nuclear divisions (MI and MII) of the synchronized meiotic cultures in (A) were assessed with fluorescence microscopy using DAPI staining to visualize nuclear division. (E) DNA replication following release from α-factor arrest was assessed via FACS and the change from 1c to 2c DNA content was plotted against time. See Figure S4 for the raw data of the FACS analysis for mitotic DNA replication. (F) The budding index of cells released from α-factor synchrony was assessed by phase contrast microscopy.

In summary, 11/81 (13.6%) of the selected genes were shown to have roles in meiotic DNA processing (Table 1). By far the highest proportion of meiotic DNA processing phenotypes 10/20 (50%) was found among mutants for genes with two or more genetic interactions (Category A). Only 1/13 (8%) from Category B, and none from Category C, D, or E conferred a meiotic DNA processing phenotype for any applied test.

### Soh1, a Component of the Mediator Complex, Bre5, a Ubiquitin Protease Co-Factor, and an Uncharacterized Protein Ygl250w Are Required for Normal Levels of Gene Conversion during Meiosis

A reduced level of gene conversion was observed for *ygl250wΔ*, *soh1Δ*, and *bre5Δ* (Figure 2B, Table 1). To further characterize these mutants and ensure efficient and synchronous initiation of meiosis, deletions were made in a sporulation-proficient S. cerevisiae strain, SK1. In this background, *bre5Δ* had a sporulation efficiency of 80% after 24 h (unpublished data); however, in pre-sporulation conditions growth of *bre5Δ* was greatly inhibited and meiosis could not be synchronized. Therefore further analysis of *bre5Δ* was not performed. For wild type, *ygl250wΔ* and *soh1Δ* pre-meiotic DNA replication, meiotic nuclear divisions, as well as molecular analyses of meiotic double-strand breaks (DSBs) and crossovers (COs), was examined. The SK1 background used carries a 3.5-kb *URA3*-*ARG4* fragment containing a recombination hotspot inserted at *his4* on one copy of Chromosome III and at *leu2* on the homologue [44]. DNA extracted from time courses of wild type, *ygl250wΔ*, and *soh1Δ* was digested with the XhoI restriction enzyme and used to assess both DSB and CO formation (Figures 2C–2E and S2).

For *ygl250wΔ*, pre-meiotic DNA replication initiates normally and progresses with similar kinetics to that of the wild type (Figure 2F), and although 15% fewer cells appear to have completed pre-meiotic DNA replication by 8 h, the level of meiotic nuclear divisions after 10 and 12 h is equivalent to the wild type (Figure 2G). DSB formation and repair during meiosis for *ygl250wΔ* also appears similar to wild type (Figure 2D), but strikingly, formation of COs was reduced by 4.5-fold (Figure 2E). For *soh1Δ*, initiation of pre-meiotic DNA replication appears to be delayed by 2 h and then proceeds with kinetics slightly below the wild type (Figure 2F). Meiotic nuclear divisions and formation of DSBs and COs are also delayed (Figure 2D, 2E, and 2G). Finally CO levels and meiotic nuclear divisions are mildly reduced compared to wild type (Figure 2E and 2G). In summary, for *ygl250wΔ* physical and genetic analysis suggests a parallel decrease in both gene conversion and CO formation, while the strong genetically determined decrease for meiotic gene conversion in *soh1Δ* was not matched by a similar lack of physical CO products in SK1.

### Def1 Is Required for Efficient Synapsis between Homologues and Normal Levels of CO Recombination during Meiosis

Six mutants in the W303 background (*bre1Δ*, *vid21Δ*, *sgf73Δ*, *rmd11Δ*, *def1Δ*, and *lge1Δ*) were found to have very low or no nuclear divisions in meiosis (Table 1). These mutants were tested to determine whether *IME1*, the master regulator of entry into meiosis [45], was properly expressed (Figure 3A). They were also tested for changes in pre-meiotic DNA replication (Figures 3B and S3), meiotic DSB formation and repair at the *THR4* hotspot (Figures 3C and S4) [46], and meiotic nuclear divisions (Figure 3D). Additionally, these six mutants showed differing levels of HU hypersensitivity (Figure S5A and Table 1); we therefore synchronized *MAT*-a cells by α-factor and monitored the progression of mitotic DNA replication by FACS (Figures 3E and S5B).

To ensure efficient and synchronous initiation of meiosis, deletions were made in the sporulation-proficient S. cerevisiae strain SK1. A synchronous culture of wild-type SK1 induces *IME1* expression and pre-meiotic DNA replication almost immediately after transfer to complete starvation conditions, and 90% of the population completes DNA replication between 5–6 h (Figures 3B and S3). Meiotic DSB formation in the wild type peaks at 4 h, and all DSBs are repaired after 7 h (Figures 3C and S4). >90% of wild-type cells have completed the first meiotic division by between 9–10 h (Figure 3D). All six mutants showed differing degrees of aberrant progression of pre-meiotic DNA replication (see below and Figure 3A–3D). For wild-type *MAT*-a cells, >90% completed DNA replication 30 min after release from α-factor, while all six mutants were slower (see below and Figure 3E and 3F).

In the SK1 background, *vid21Δ* grew very slowly under pre-meiotic growth conditions and hardly showed any induction of *IME1* expression (Figure 3A). Furthermore, pre-meiotic DNA replication and meiotic nuclear divisions were not detected (unpublished data).

In the *rmd11Δ* strain, the induction of *IME1* was normal (Figure 3A); however DNA replication started with a delay of approximately 3 h, but proceeded with normal speed thereafter. Meiotic DSB formation and nuclear divisions occurred with a similar delay and also proceeded with fairly normal speed, but disappearance of DSBs was greatly delayed (Figure 3C and 3D), suggesting problems in DSB repair. Notably, in contrast to the SK1 background, a strong reduction in nuclear divisions had been observed for *rmd11Δ* in the W303 background (Figure 3D and Table 1). From α-factor synchronization a delay in G1 to S-phase transition was also observed for *rmd11Δ* (Figure 3E). Interestingly, according to the budding index, *rmd11Δ* does not affect the rate of bud formation (Figure 3F).

The remaining four mutants all were impaired for normal progression of pre-meiotic DNA replication. The *bre1Δ* and *lge1Δ* mutants showed normal induction of *IME1* (Figure 3A). However, *bre1Δ* and *lge1Δ* started with a delay in pre-meiotic DNA replication, and 90% of the population completed DNA replication between 22–24 h (Figure 3B). Interestingly, the levels of DSBs formed in the sporulating *bre1Δ* and *lge1Δ* populations were reduced and the majority of them appeared to be repaired after 11–12 h (Figure 3C). Additionally, meiotic nuclear divisions were strongly reduced for *bre1Δ* and *lge1Δ* reaching only 57% and 52% after 48 h, respectively (Figure 3D). The *sgf73Δ* and *def1Δ* strains did not show a clear delay in entry into pre-meiotic DNA replication; however, they did show a lengthened time to complete pre-meiotic DNA replication (Figure 3B). Additionally, less than 80% of the population for both strains completed pre-meiotic DNA replication. The meiotic nuclear divisions for *sgf73Δ* and *def1Δ* were also strongly reduced reaching 27.5% and 35% after 48 h, respectively. The level of *IME1* induction observed for *sgf73Δ* was reduced to 40% of wild-type levels (Figure 3A). The low level of *IME1* induction could explain why *sgf73Δ* showed slow progression of pre-meiotic DNA replication and inefficient meiotic nuclear divisions. Additionally, *def1Δ* showed a mild reduction to roughly 70% of wild-type levels of *IME1* induction (Figure 3A). However, this reduction does not explain the strength of the observed phenotypes in *def1Δ*. All four mutants also show a clear delay in initiation and progression of mitotic DNA replication (Figure 3E). In addition, FACS profiles show that 35% of *def1Δ* cells failed to enter G1 during α-factor synchronization (Figure 3E) and 11% of this population contained >2c (copies of the genome) DNA content. This may be partly due to defective cytokinesis after nuclear division, as 20% of *def1Δ* cells develop multiple large buds that sometimes contain more than a single nucleus.

The six mutations were also assessed in a *spo11Δ*, *spo13Δ* background to determine whether their sporulation phenotypes could be bypassed in the absence of meiotic recombination. Only *def1Δ* showed improvement of sporulation efficiency; however, spore viability was not improved in the absence of meiotic recombination (unpublished data).

**Figure 4:**
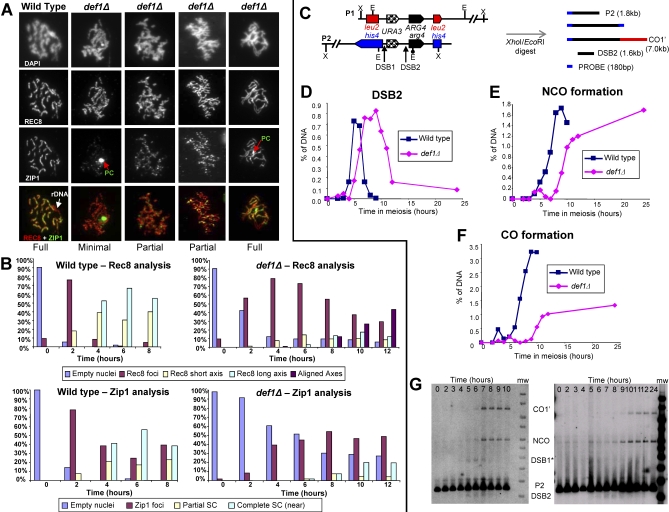
Assessment of *def1*Δ in Meiosis and Mitosis (A) Immunocytology of nuclear spreads of SK1 wild-type and *def1Δ* strains after 8 h of sporulation. The meiosis-specific subunit of cohesin, Rec8, was tagged with multiple Haemagglutinin (HA) epitopes. Using antibodies for HA and Zip1 allowed analysis of sister chormatid cohesion and synaptonemal complex formation, respectively. It can be seen in the wild-type example that all 16 chromosomes have long cohesin axes and close to full chromosome synapsis except for the rDNA region on Chromosome XII. Whereas from the first panel for *def1Δ* it can be seen that axes are aligned but synapsis is minimal. The second and third panels for *def1Δ* again show aligned axes, but homologues are only partially synapsed. However, as shown in the final panel, synapsis was observed in some meiotic nuclei of the *def1Δ* strain. Polycomplexes (PCs) of Zip1 were observed in 20% of the nuclei counted for *def1Δ* at this time point whereas less than 1% PCs were observed for the wild type. (B) Time course of the meiotic nuclei counted using immunocytology for both wild type and *def1Δ* during meiosis. The *def1Δ* mutant synapsis phenotype represented in (A) was counted as “aligned” axes in the Rec8 analysis graph. At least 200 nuclei were counted per time point. (C) Ectopic *URA3*-*ARG4* interval on Chromosome III described in Figure 3. XhoI and EcoRI restriction sites are indicated by “X” and “E,” respectively. To detect NCOs, COs, and DSBs, DNA is digested with XhoI and EcoRI then probed with HIS4 sequences (hisU; [44]). For graphs (D–F), wild-type and *def1Δ* are represented by blue squares and pink diamonds, respectively. The corresponding XhoI and EcoRI double digest Southern blots, the XhoI single digest Southern blots, together with the molecular analyses, are presented in Figure S5. (D) Molecular analysis for DSB (DSB2) signal/total lane signal from Southern blots of DNA extracted from synchronized meiotic cultures. (E) Molecular analysis for NCO (NCO1) signal/total lane signal from Southern blots of DNA extracted from synchronized meiotic cultures. (F) Molecular analysis for CO (CO1') signal/total lane signal from Southern blots of DNA extracted from synchronized meiotic cultures. (G) Southern blot of DNA isolated from wild-type and *def1Δ* SK1 strains containing the ectopic *URA3*-*ARG4* interval on Chromosome III. DNA was digested with XhoI and EcoRI then probed to detect NCOs, COs, and DSBs; mw represents the 1-kb molecular weight marker (Fermentas).

To analyze sister chromatid cohesion and homologue synapsis in these mutants, meiotic nuclear spreads of each strain were immunostained for Rec8, the meiosis-specific cohesin subunit, and Zip1, a synapsis-specific component of the synaptonemal complex. As expected from the pre-meiotic DNA replication data, all strains showed a delay in the formation of Rec8 axes and were late in chromosome synapsis, with the exception of *vid21Δ*, which did not show axis formation or chromosome synapsis. The other five strains frequently contained Zip1 polycomplexes, an indication of a delay of synapsis relative to Zip1 expression (unpublished data). With the exception of the Zip1 polycomplexes, the majority of nuclei observed for the *rmd11Δ*, *sgf74Δ*, *bre1Δ*, and *lge1Δ* at later time points displayed normally synapsed chromosomes. However, nuclei containing long Rec8 axes and full synapsis were greatly reduced in *sgf74Δ*, *bre1Δ*, and *lge1Δ* mutants (unpublished data). The *def1Δ* strain showed interesting defects in chromosome morphology indicating uncoupling of axis formation from synapsis (Figure 4A and 4B). These events occur in parallel in wild type and also in most mutants with delayed synapsis. The *def1Δ* strain was strongly delayed for the formation of Rec8 axes showing lack of condensation, probably owing to the slow replication despite relatively normal Rec8 expression. Synapsis was strongly reduced and hardly detectable before 8 h in sporulation media. Even if long condensed Rec8 axes were formed and pairwise-aligned by one or more axial association sites, synapsis frequently did not commence, a situation not occurring in wild type (Figure 4A). Similar observations have been made for mutants of the ZMM class of meiotic genes (*ZIP1*, *ZIP2*, *ZIP3*, *ZIP4*/*SPO22*, *MSH4*, *MSH5*, and *MER3*) that are directly involved in initiation and progression of synapis [33,47–50]. A hallmark of these ZMM mutants is the specific reduction in CO, without affecting noncrossover (NCO) recombination during meiosis. Therefore, molecular analysis of the level of CO and NCO recombination in *def1Δ* was assessed. For this, a mutation of *DEF1* was created in a SK1 strain that carries a 3.5-kb *URA3*-*ARG4* recombination interval inserted at *his4* on one copy of Chromosome III and at *leu2* on the homologue (Figure 4C) [44]. DNA extracted from synchronized sporulation time courses of wild type and *def1Δ* was digested with the XhoI and EcoRI restriction enzymes and used to assess DSB, NCO, and CO formation (Figures 4C–4G, S6A, and S6B). As observed for the *THR4* hotspot (Figure 3C), DSB repair in *def1Δ* appears to take longer than in the wild type (Figure 4D and 4G). As a result, the appearance of CO and NCO recombination products are also delayed (Figure 4E–4G). Strikingly, the formation of COs is reduced in *def1Δ* to 35% of wild-type levels (Figure 4F), whereas NCO levels are largely unaffected (Figure 4E). Furthermore, as described in Figure 3C, DNA from the wild-type and *def1Δ* sporulation time courses were also digested with XhoI to assess meiotic DSBs and COs. Again DSB repair was delayed, and CO formation was reduced in *def1Δ* (Figure S6C–S6H). Thus, as predicted by the cytological phenotype, *def1Δ* is specifically defective in Zip1 assembly and CO control, identifying *DEF1* as a ZMM gene.

### Deletion of PMR1 Causes HU Sensitivity and Formation of Multads in Meiosis

Mutation of *HUR1* has been reported to cause increased sensitivity to HU [51]. However, *HUR1* partially overlaps with *PMR1*, a gene that encodes an ATPase required for Ca^2+^ and Mn^2+^ import into the Golgi apparatus (Mandal, et al. 2003). Therefore mutations that interrupt sections of the open reading frames of *HUR1* and *PMR1* separately were created (Figure S7A). This analysis revealed that deletion of *PMR1* but not *HUR1* affected resistance to HU (Figure S7B). FACS analysis showed that *PMR1* is required for normal timing of initiation and progression of DNA replication during mitosis (Figure S7C). Additionally *pmr1Δ* cells formed some abnormal “multad” asci containing more than four inviable spores (Figure S7D). The sporulation efficiency of *pmr1Δ* was 55%, and up to 52% of the asci contained >4 spores. Analysis of meiotic DNA replication in the *pmr1Δ* strain revealed that after 12 h, 30% of the cells had a DNA content greater than four copies of the genome suggesting rereplication or lack of cytokinesis prior to meiosis as the basis for multad formation (Figure S7E).

## Discussion

### Systematic Integration of High-Throughput Data

Although high-throughput experiments have provided insight into gene function, it has also become apparent that single datasets have limitations. False positive data are common. For yeast two-hybrid data it has been estimated that only 50% of the reported interactions are of biological relevance [27]. It is known that gene epitope tagging can result in incorrect protein localization data [20,21]. Additionally, 6.5% of the yeast genome deletion library is problematic with respect to background mutations [26]. Procedures used for high-throughput experiments can also give rise to limitations. For example, protein localization analyses have been performed in vegetative cells under normal growth conditions, whereas a number of proteins may only localize when exposed to a certain environmental condition. For yeast two-hybrid interaction experiments, the “bait” and “prey” proteins interact inside the nucleus, which in many cases is not their native cellular compartment. Protein complex purification experiments are biased towards proteins that are of high abundance [34].

Due to these limitations, a number of methods have been developed to combine datasets to determine whether the data support each other. Methods have been used to combine mRNA expression with protein interaction data [29,36,38] and from these studies it was found that proteins that interact often have a correlation in mRNA expression pattern. More recently, work combining mRNA expression, genetic interactions, and database annotations was used to validate protein interaction data [52].

Recently researchers have begun to develop a number of in-silico methods to predict gene function by integrating a number of high-throughput datasets [52–56]. However, to our knowledge only three integration methods include the high-throughput genetic interaction datasets for S. cerevisiae [52,55,57]. These studies either provide very little or no experimental analysis of their predictions [52,55,57]. Our data mining was based on the knowledge acquired from previous data integration techniques to set the selection criteria (Figure 1), and we have set out to test the predictions in experimental detail.

Our selection strategy identified 81 genes of which 16 (20%) caused at least one irregular DNA processing phenotype when mutated. Interestingly, all but one of these selected genes had at least one genetic interaction with a DNA processing gene. In an aim to avoid false candidates we saw fit to exclude 13 genes because the fraction of their interaction partners involved in DNA processing was less than 1/5. However, four of these genes have now been shown to have a role in DNA processing. Therefore this selection step was not beneficial.

### Genes Required for Normal Levels of Meiotic Gene Conversion and CO Formation during Meiosis

Three genes were found to be required for normal levels of gene conversion during meiosis, *SOH1*, *BRE5*, and *YGL250W*.

(1) *SOH1* was first discovered as a gene that suppressed the hyper-recombination phenotype of *hpr1Δ* [58]. Hpr1 is a component of the THO/TREX complex which couples transcription elongation with mitotic recombination [59]. Soh1 was later shown to be a component of the Mediator complex [60], which is required to stimulate gene transcription by transmission of regulatory signals from transcription activators to RNA polymerase II during stress responses [61]. *SOH1* has a number of genetic interactions with genes required for DNA replication (e.g., *RAD52*, *RAD50*, *RAD55*, and *RAD6*), DNA repair (e.g., *CDC45*, *MRC1*, and *ORC2*) and chromatid cohesion (e.g., *CTF4*, *CTF8*, and *CTF18*), and the *soh1Δ* mutant was observed here to have sensitivity to HU in addition to reduced gene conversion levels, whilst not greatly affecting CO formation during meiosis. Our observations suggest that the Mediator complex also has a role in regulation of DNA replication and recombination.

(2) Bre5 is a conserved protein that has been shown to form a complex with ubiquitin protease Ubp3 that is required for the de-ubiquitination of subunits of coat protein complexes I and II that are involved in transport between the endoplasmic reticulum and Golgi apparatus [62,63]. *BRE5* has been reported to have genetic interactions with genes involved in cell wall organization and biogenesis [13]. However, here the gene was selected for its genetic interactions with DNA processing genes [8,13]; therefore, it is conceivable that *BRE5* functions in a number of cellular pathways, one of which is DNA processing. In this study, *BRE5* was shown to be required for normal levels of sensitivity to HU and meiotic gene conversion. Perhaps Bre5 is required for de-ubiquitination of proteins required for DNA replication/recombination. Due to the slow growth phenotype of *bre5Δ* in pre-sporulation conditions, a meiotic-specific null allele would be required to assess its role in meiotic DNA processing more closely.

(3) *YGL250W/RMR1* (named here Reduced Meiotic Recombination 1) was shown to be required for normal levels of gene conversion and CO formation during meiosis. This gene has been reported to have synthetic lethal interactions with both *CDC7* and *MCD1/SCC1* [13]. Interestingly, Cdc7-Dbf4 is required for recombination, synaptonemal complex formation, and chromosome segregation during meiosis [64,65]. Additionally, Mcd1/Scc1 is a subunit of the cohesin complex which is required for sister chromatid cohesion in mitosis and meiosis [66,67]. The meiotic DNA processing role of *RMR1* remains to be determined. However Rmr1 appears to be sumoylated [68], which could be important for its function.

### Genes Required for Normal Progression of Pre-meiotic DNA Replication

Six mutants sensitive to HU during vegetative proliferation also displayed reduced nuclear division in meiosis. These mutants were found to impair mitotic and meiotic DNA replication.

(1) The *vid21Δ* strain was found to be sensitive to X rays and MMS and the gene was required for detectable expression of *IME1* and initiation of pre-meiotic DNA replication. Since Ime1 is required for the initiation of meiotic events including pre-meiotic DNA replication [69], lack of Ime1 induction is sufficient to explain the phenotypes. Vid21 was recently identified as a novel component of the histone acetyltransferase NuA4 and is required for bulk H4 histone acetylation [70]. Other components of NuA4 are also required for maintenance of DNA integrity [70]. A mutant for another NuA4 subunit (Yng2) was found not to progress through meiosis [71], however expression of Ime1 and pre-meiotic DNA replication were not assessed. The chromatin remodelling Swi/Snf complex is required for high level expression of *IME1* [72]. H4 histone acetylation is associated with transcriptional induction, and it is conceivable that NuA4 directly up-regulates *IME1* and other early meiotic genes upon sporulation. However, a meiotic phenotype for other components of the histone acetyltransferase has not been reported.

(2) Recently, Sgf73 was found to be a component of two histone acetyltransferases, namely SAGA and SLIK [73], which are both required for gene expression. Here the *SGF73* mutant showed abnormal pre-meiotic DNA replication, and expression of *IME1* was reduced. This reduced *IME1* expression may account for the observed meiotic phenotypes. In addition to our observation that *sgf73*Δ is hypersensitive to HU, it has been shown that Sgf73 is required for the recruitment of the SAGA factor to upstream activating sequences that facilitates formation of the replication pre-initiation complex [74], thus confirming a direct role in DNA replication.

(3) Prior to this work, it was known that *BRE1* and *LGE1* are required for ubiquitination of histone H2B and K3 methylation of H4 during vegetative proliferation [75]. However, their effects during meiosis had not been reported. Here we show that *lge1Δ* and *bre1Δ* strains are characterized by delayed initiation of meiotic DNA replication and lengthened time for completion. Recently, *bre1Δ* was shown to affect pre-meiotic DNA replication onset and progression, as well as DSB formation. That work found that Bre1 is an E3 ubiquitin ligase that exists as a complex with the E2 ubiquitin-conjugating enzyme Rad6 [76]. The Bre1-Rad6 complex was shown to ubiquitinate lysine 123 of histone H2B, which is required for normal levels and timing of DSB formation during meiosis. Here delay of onset and slowed progression of pre-meiotic DNA replication and reduced levels of meiotic DSBs for *bre1Δ* were also observed. Furthermore, the *lge1Δ* strain was observed to have the similar pre-meiotic S-phase pattern and reduction in meiotic DSB formation as the *bre1Δ* strain. As Lge1 co-purifies with Bre1 during vegetative proliferation [6,75], we predict that Lge1 may also function with Bre1 during meiosis. However, it should be noted that Lge1 does have a mitotic function that is independent to Bre1 and Rad6. In cells that have lost their mitochondrial genome, Lge1 is required for the induction of *PDR3* and *PDR5* expression which are both involved in multidrug resistance [77].

(4) Def1 forms a complex with Rad26 and recruits the E3 ubiquitin ligase Rsp5 to sites of DNA damage to ubiquitinate stalled RNA polymerase II to mark it for degradation [78–80]. However, only a minor fraction of the protein associates with Rad26 via immunoprecipitation [80], raising the possibility of other cellular roles. In fact, independent of its role with Rad26, Def1 was found to be required for telomere maintenance and shown to physically interact with Rrm3, a helicase required for replication of DNA at the telomeres [81]. Here we have shown that in addition to the sensitivity of *def1Δ* to HU, MMS, and X rays, both vegetative and pre-meiotic DNA replication are strongly affected. *def1Δ* also displays a prominent defect in the synapsis of homologous chromosomes during meiosis. Paired, but only loosely connected, chromosomes were observed, in which chromosome axes were fully formed, but synapsis had not commenced. This phenotype was also observed in mutants deficient for Zip1 loading (ZMM group) [33,47–50], some of which show similarity to components of the APC, a multisubunit ubiquitin ligase [21]. Interestingly, *def1Δ* shows the specific reduction in COs, without affecting NCO recombination, which is another hallmark of ZMM mutants. From chromatin immunoprecipitation data, Def1 has been shown to bind to both telomeric and non-telomeric DNA [81]. Therefore it will be of interest to assess whether Def1 also binds to DNA during meiosis, and furthermore if its localization is correlated to its apparent requirement for efficient synapsis.

(5) *RMD11* was selected in this study due to a reported synthetic lethal interaction with *cdc45–1Δ* [13] and a protein interaction with Dcc1 [2]. Prior to the completion of this work, *RMD11* was also shown to have synthetic sick interactions with *POL32* and *CSM3* [11]. Therefore, these interactions associate Rmd11 with the biological process of DNA replication. *RMD11* (Required for Meiotic Nuclear Divisions) was reported to be essential for sporulation but not to be required for *IME1* induction [22]. Here we confirmed these data for the W303 background, but showed that *RMD11* is not essential for meiosis in the efficiently sporulating SK1 strain background. In the SK1 background, pre-meiotic DNA replication was delayed, but eventually spores formed and were largely viable. Additionally, initiation of DNA replication during vegetative growth was delayed, suggesting that *RMD11* is required for the efficient initiation of DNA replication. Furthermore, *rmd11Δ* was found to have an increased sensitivity to HU, which slows or inhibits DNA replication. Interestingly, Rmd11 is a member of an uncharacterized protein family that includes members in many model organisms as well as Homo sapiens.

(6) In addition to HU hypersensitivity, *pmr1Δ* was found by FACS analysis to affect pre-meiotic DNA replication and result in the formation of asci with more than four inviable spores. Pmr1 is an ATPase required for Ca^2+^ and Mn^2+^ transport into the Golgi [82]. However, *PMR1* has genetic interactions with genes involved in DNA replication (e.g., *POL32* and *RRM3*), DNA repair (e.g., *MRE11*, *RAD55*, *RAD51*, *RAD18, MMS1*, and *RTT107*), and chromatid cohesion (e.g., *DCC1*, *CTF4*, and *CTF18*), and the mutant phenotypes observed in this study suggest that *PMR1* also plays a role in DNA processing.

### Conclusions

A strategy of integrating high-throughput data can be successfully used to imply a role in DNA processing for minimally characterized genes. Genetic interaction data have proved to be extremely valuable in the success of our selection strategy. This feature encourages further genetic interaction analyses to be performed not only in yeast, but in all model organisms.

Among the 16 genes identified to be involved in DNA processing, 11 had a role in meiotic DNA processing, including *DEF1*, which was found to be required for efficient chromosome synapsis and specific reduction in CO, without affecting NCO recombination during meiosis. In addition, three genes (*SOH1*, *BRE5*, and *YGL250W/RMR1*) were found to be required for normal levels of meiotic gene conversion and three genes (*YPL017C*, *SOH1*, and *MMS22*) required for accurate chromosome segregation during meiosis.

## Materials and Methods

### Parent and deletion strains.

All strains used in this work are presented in Table S4. Deletion strains were transformed with PCR-generated disruption cassettes containing the *KANMX4* marker gene [83,84]. Gene deletions were confirmed by PCR for three clones of each transformation.

### Mitotic DNA processing screens.

Spot plates were prepared on YPD (control) YPD + 100 mM HU (Sigma), YPD + 0.03% MMS (Sigma), YPD exposed to 40 min of X ray 120 kVp (Torrex cabinet X-ray system, Faxitron X-ray Corporation).

### Molecular analyses.

Meiotic DSBs and recombination products were detected by Southern blotting using ^32^P-ATP- (GE Healthcare) labeled DNA probes. Signals were detected using the Storm Phosphoimager (GE Healthcare) and blots were quantified using ImageJ version 1.37 [85]. The methods used for the physical analysis of DSBs at the *THR4* hotspot have been previously described [46]. The methods used for the physical analysis of DSBs, COs, and NCOs of the diploid strains that carry a 3.5-kb *URA3*-*ARG4* recombination interval inserted at *his4* on one copy of Chromosome III and at *leu2* on the homolog have been previously described [44].

### Cytology.

Yeast meiotic spreads were performed as described [67,86]. Rec8-HA was detected using 16B12 (mouse anti-HA, 1:1,600) and CY3-conjugated goat anti-mouse antibody (1:200, Dianova). Rabbit anti-Zip1 antibody was raised against a purified Zip1-GST fusion protein and affinity purified against the same protein. The purified Zip1 antibody was used (1:50) and detected by fluorescein isothiocyanate (FITC)-conjugated goat anti-rabbit serum (1:200, Sigma).

### Cell synchrony and analysis of DNA replication.

Haploid *MAT*-a cells were synchronized in G1 with α-factor using a method previously described [87]. SK1 diploids were synchronized for meiosis using a method previously described [88]. Cells were prepared for FACS analysis using a method previously described [89] and observed using a FACSCalibur (BD Biosciences) and CellQuant software, version 3.3 (BD Biosciences).

### Random spore analysis for meiotic gene conversion and chromosome missegregation assays.

Sporulation efficiency was determined for W303 diploids and the equivalent of 5 × 10^8^ tetrads were digested with 100 μg/ml Zymolyase (Zymo Research). Single spores were prepared as previously described [90] and were plated onto SC without arginine but plus canavanine to select for haploid can^R^ cells. To test for gene conversion, lysine was omitted, and to assess missegregation, adenine was omitted.

## Supporting Information

Dataset S1 Gene Accession Numbers (21 KB XLS)

Figure S1 Examples of Selected Genes with an Implied Role in DNA Processing Genes are represented as nodes and interactions are represented as lines (edges) that connect the nodes with an arrow signifying the direction from the query gene/protein to the interacting gene/protein [91]. Gene function and interaction type is signified by the colour scheme described in the figure key. The red numbers represent the gene expression correlation distances calculated for all physical interaction data [29].
(1–3) Category A: *YPL017C*, *PMR1* (*HUR1*), and *YGL250W* are examples of genes having two or more genetic interactions reported with DNA processing genes.
(4) Category B: *RMD11* is an example of a gene that has one genetic interaction and one or more physical interaction(s) with gene expression correlation distance below the cut-off value set (<0.9) with DNA processing genes.
(5) Category C: *YGL071C* is an example of a gene that has one genetic interaction and one or more physical interactions(s) without gene expression correlation distance above the cut-off value of 0.9.
(6) Category D: *PSY3* is an example of a gene that has two or more physical interactions that have gene expression correlation distances with DNA processing genes below our cut-off value of 0.9.
(7) Category E: *YMR233W* is an example of a gene that has less than two interactions that have gene expression correlation distances with DNA processing genes below the cut-off value of 0.9.
See Table S3 for interaction data of all 81 genes selected.
Following the selection of these genes, additional interactions with DNA processing genes have since been reported. Additional interaction data for the 81 genes selected have been entered into Table S3. Also see the Yeast General Repository for Interaction Datasets (GRID) [30].
(54 KB PPT)

Figure S2 Southern Blot of DNA Isolated from (A) Wild Type, (B) *soh1Δ*, and (C) *ygl250wΔ* SK1 Strains Containing the Ectopic *URA3*-*ARG4* Interval on Chromosome III (D) The DNA from the indicated times after initiation of sporulation were digested with XhoI then probed to detect COs and DSBs; mw1 represents the λ-HindIII molecular weight marker (Fermentas) and mw2 represents the 1-kb molecular weight marker (Fermentas).
(465 KB DOC)

Figure S3 FACS Analysis of Pre-Meiotic DNA Replication for VID21, BRE1, LGE1, RMD11, SGF73, and DEF1 Mutants Raw output from FACS analysis of each SK1 strain synchronized for entry into the meiotic cell cycle. Cells were counted at a rate between 250–280 cells per s. FACSCalibur apparatus and CellQuant Version 3.3 (BD Biosciences) were used for analysis.
(383 KB DOC)

Figure S4 Southern Blot Analysis of DSB Formation during Meiosis for *BRE1*, *LGE1*, *RMD11*, and *DEF1* Mutants (A) Schematic representation of the natural *THR4* meiotic DSB hotspot on Chromosome III. DNA from synchronized sporulation time courses of each strain were cut with the BglII restriction enzyme, and using a probe upstream to *THR4*, presence of DSBIII and DSBIV (indicated by the arrows) together with the parental were assessed [87]. Southern blots of wild type (B), *rmd11Δ* (C), *def1Δ* (D), *bre1Δ* (E), and *lge1Δ* (F). The DNA from the indicated times after initiation of sporulation were digested with BglII then probed to detect DSBIII and DSBIV from the *THR4* hotspot; mw represents the 1-kb molecular weight marker (Fermentas).
(509 KB DOC)

Figure S5 Analysis of Mitotic DNA Replication for *VID21*, *BRE1*, *LGE1*, *RMD11*, *SGF73*, and *DEF1* Mutants (A) Overnight cultures were diluted in series by a factor of 10 from 10^−2^ to 10^−5^ and plated on YPD and YPD + 100 mM HU. Mutants were defined as having increased sensitivity or decreased viability to HU in comparison to the wild type.
(B) FACS analysis of each *MAT*-a strain synchronized in G1 with α-factor and then released into S phase. Cells were counted at a rate between 250–280 cells per s. FACSCalibur apparatus and CellQuant Version 3.3 (BD Biosciences) were used for analysis.
(664 KB DOC)

Figure S6 Assessment of *def1Δ* in Meiosis Southern blot of DNA isolated from SK1 wild-type (A) and *def1Δ* (B) strains containing the ectopic *URA3*-*ARG4* interval on Chromosome III (Figure S2). The DNA from the indicated times after initiation of sporulation were digested with XhoI and EcoRI then probed to detect NCOs, COs, and DSBs; mw1 represents the λ-HindIII molecular weight marker (Fermentas) and mw2 represents the 1-kb molecular weight marker (Fermentas).
Southern blot of DNA isolated from SK1 wild-type (C) and *def1Δ* (D) strains containing the ectopic *URA3*-*ARG4* interval on Chromosome III described in Figure 3. The DNA from the indicated times after initiation of sporulation were digested with XhoI then probed to detect COs and DSBs; mw1 represents the λ-HindIII molecular weight marker (Fermentas) and mw2 represents the 1-kb molecular weight marker (Fermentas).
(E) Molecular analysis for DSB (DSB1 + DSB2) signal/total lane signal from Southern blots of DNA extracted from synchronized meiotic cultures.
(F) Molecular analysis for CO1 signal/total lane signal from Southern blots of DNA extracted from synchronized meiotic cultures.
(G) Molecular analysis for CO2 signal/total lane signal from Southern blots of DNA extracted from synchronized meiotic cultures.
(H) Molecular analysis for CO (CO1 + CO2) signal/total lane signal from Southern blots of DNA extracted from synchronized meiotic cultures.
(676 KB DOC)

Figure S7 Assessment of *pmr1Δ* (and *hur1Δ*) in Mitosis and Meiosis (A) Diagram showing the 181-bp overlap between the open reading frames for *HUR1* and *PMR1* on Chromosome VII; two *KANMX4* gene deletion mutants were constructed using cassettes that only interfere with either *HUR1* (1–81 bp of *HUR1* ORF, *hur1Δ1–81*, represented by black region) or *PMR1* (1–2,233 bp of *PMR1* ORF, *pmr1Δ1–2,233*, represented by grey region). The arrows on each strand represent direction of transcription.
(B) HU sensitivity assay using the *hur1Δ1–81* and *pmr1Δ1–2,233* strains shows that *PMR1* and not *HUR1* is required for resistance to HU.
(C) FACS analysis of cells released from α-factor synchrony shows that the progression of mitotic DNA replication is slowed in *pmr1Δ1–2,233*.
(D) Microscopy of sporulation sample of SK1 wild type and *pmr1Δ1–2,233* using differential interference contrast (DIC) and fluorescence microscopy to view segregation of Chromosome V (tagged with green fluorescent protein). The SK1 *pmr1Δ1–2,233* mutant not only gives rise to tetrads, but also “multads” that contain greater than four spores.
(E) DNA replication during meiosis was assessed via FACS. 35% of the population of the *pmr1Δ1–2,233* cells have a DNA content that is greater than a single round of diploid DNA replication (>4c) observed in the wild type. The *pmr1Δ1–2,233* strain does not grow well during pre-meiotic conditions; therefore the FACS analysis experiment was not optimal.
(428 KB DOC)

Table S1 Click here for additional data file. List of DNA Processing Genes Used for Selection
(124 KB XLS)

Table S2 Click here for additional data file. List of Genes Removed during the Secondary Selection
(474 KB XLS)

Table S3 Click here for additional data file. List of Five Subdivided Categories of the 81 Genes Selected for Analysis
(175 KB XLS)

Table S4 Click here for additional data file. List of Strains Used in This Work
(315 KB XLS)

## Abbreviations

c: copies of the genome
CO: crossover
DSB: double-strand break
HU: hydroxyurea
MMS: methanesulphonate
NCO: noncrossover
