# Supplementary material for: Novel Roles for Selected Genes in Meiotic DNA Processing
Source: PLoS Genet. 2007 Dec 7;3(12):e222. doi: 10.1371/journal.pgen.0030222 (PMC2134943; doi:10.1371/journal.pgen.0030222)
Supplement: Supplementary file 7 [file pgen.0030222.st004.doc]

Supplementary Table S4.1

Saccharomyces cerevisiae Parental Strains

| Strain | Accession Number | Genotype | Source |
| --- | --- | --- | --- |
|  |  |  |  |
| BY4743 | Y2 | MATa*/*MAT*α, his3Δ1/his3D1, leu2Δ0/leu2Δ0, met15Δ0/MET15, LYS2/lys2Δ0, ura3Δ0/ura3Δ0, hoΔ0* | EUROSCARF |
|  |  |  |  |
| SK1 | FK2 | MATa*/*MAT*α, HO* | F. Klein,  Chromosome Biology, Vienna, Austria |
|  |  |  |  |
| K8409  (SK1 derivative) | K2 | MATa*/*MAT*α, HO, promURA3::tetR::GFP-LEU2, tet0x224-URA3, REC8-HA3::URA3, ADE2, his3::hisG, trp::hisG* | A. Toth,  IMP, Vienna, Austria |
|  |  |  |  |
| K4387  (SK1 derivative) | N2 | MATa*/*MAT*α, ho::LYS2, lys2Δ0, leu3::hisG, his4Δ0, ura3Δ0, +* *pAS128* (*proIME1-lacZ*) | F. Klein,  Chromosome Biology, Vienna, Austria |
|  |  |  |  |
| FK3035  (SK1 derivative) | M0 | MAT*α, ho::LYS2, lys2Δ0, arg4(Eco47III-hpa1), leu2-R, ura3, his4Δ (Sal1-Cla1)::URA3-Δ(Sma1-Eco47III)-arg4-EcPal(1691)* | M. Lichten,  Centre for Cancer Research, Maryland, USA |
|  |  |  |  |
| FK3036  (SK1 derivative) | M1 | MATa, *ho::LYS2, lys2Δ0, arg4(Eco47III-hpa1),cyh2-z, ura3, leu2-RV::URA3-(Sma1-Eco47III)-[ARG4cloned]* | M. Lichten,  Centre for Cancer Research, Maryland, USA |
|  |  |  |  |
| FK192  (SK1 derivative) | S0 | MAT*α, spo11::URA3, spo13::hisG, leu2-3, trp1::hisG, ura3-1* | F. Klein,  Chromosome Biology, Vienna, Austria |
|  |  |  |  |
| FK193  (SK1 derivative) | S1 | MATa*, spo11::URA3, spo13::hisG, leu2-3, trp1::hisG, ura3-1* | F. Klein,  Chromosome Biology, Vienna, Austria |
|  |  |  |  |
| Table S4.1 *Saccharomyces cerevisiae* Parental Strains (Continued) | | | |
| Strain | Accession Number | Genotype | Source |
|  |  |  |  |
| EUF153  *rad5-G535R*  (W303 derivative) | R0 | MATa*, his3-11, leu2-3, -112, ura3-1, trp1-2Δ*  *ade2-1, ade1::ADE2, can1-100, lys2-nde3’,*  *rad5-G535R* | R. Borts,  University of Leicester, England |
|  |  |  |  |
| EUF180  *rad5-G535R*  (W303 derivative) | R1 | MAT*α, his3-11, leu2-3, -112, URA3, trp1-2Δ*  *ade2-1, ADE1, CAN, lys2-nde5’, rad5-G535R* | R. Borts,  University of Leicester, England |
|  |  |  |  |
| PKY 781  (W303 derivative) | P0 | MATa*, leu2-3, ura3-1, his3-11, trp1-1, ade2-1, can1-100, RAD5* | P. Kaufman,  University of California, USA |
|  |  |  |  |
| PKY 782  (W303 derivative) | P1 | MAT*α,, leu2-3, ura3-1, his3-11, trp1-1, ade2-1, can1-100, RAD5* | P. Kaufman,  University of California, USA |
|  |  |  |  |
| EUF153  (W303 derivative) | E0 | MATa*, his3-11, leu2-3, ura3-1, trp1-2Δ*  *ade2-1, ade1::ADE2, can1-100, lys2-nde3’,*  *RAD 5^+^* | This work |
|  |  |  |  |
| EUF180  (W303 derivative) | E1 | MAT*α, his3-11, leu2-3, URA3, trp1-2Δ*  *ade2-1, ADE1, CAN, lys2-nde5’, RAD 5^+^* | This work |
|  |  |  |  |

**Supplementary Table S4.2**

*Saccharomyces cerevisiae* KANMX4 Deletion Strains

| Gene Replaced (Gene number) | Parent Strains | | | |
| --- | --- | --- | --- | --- |
|  |  |  |  |  |
| YAL019W  (0389) | BY4743 | K8409 | EUF153-RAD5^+^ | EUF180-RAD5^+^ |
|  |  |  |  |  |
| YAL027W  (0381) | BY4743 | K8409 | EUF153-RAD5^+^ | EUF180-RAD5^+^ |
|  |  |  |  |  |
| YAL034C  (0374) | BY4743 | K8409 | EUF153-RAD5^+^ | EUF180-RAD5^+^ |
|  |  |  |  |  |
| YBL031W  (3057) | BY4743 | K8409 | EUF153-RAD5^+^ | EUF180-RAD5^+^ |
|  |  |  |  |  |
| YBR025C  (3162) | BY4743 | K8409 | EUF153-RAD5^+^ | EUF180-RAD5^+^ |
|  |  |  |  |  |
| YBR094W  (3233) | BY4743 | K8409 | EUF153-RAD5^+^ | EUF180-RAD5^+^ |
|  |  |  |  |  |
| YBR095C  (3234) | BY4743 | K8409 | EUF153-RAD5^+^ | EUF180-RAD5^+^ |
|  |  |  |  |  |
| YBR125C  (7163) | BY4743 | K8409 | EUF153-RAD5^+^ | EUF180-RAD5^+^ |
|  |  |  |  |  |
| YBR129C  (3268) | BY4743 | K8409 | EUF153-RAD5^+^ | EUF180-RAD5^+^ |
|  |  |  |  |  |
| YBR184W  (3324) | BY4743 | K8409 | EUF153-RAD5^+^ | EUF180-RAD5^+^ |
|  |  |  |  |  |
| YBR225W  (3365) | BY4743 | K8409 | EUF153-RAD5^+^ | EUF180-RAD5^+^ |
|  |  |  |  |  |
| YBR231C  (3371) | BY4743 | K8409 | EUF153-RAD5^+^ | EUF180-RAD5^+^ |
|  |  |  |  |  |
| YBR270C  (7168) | BY4743 | K8409 | EUF153-RAD5^+^ | EUF180-RAD5^+^ |
|  |  |  |  |  |
| YDL070W  (3767) | BY4743 | K8409 | EUF153-RAD5^+^ | EUF180-RAD5^+^ |

| Table S4.1 *Saccharomyces cerevisiae* *KANMX4* Deletion Strains (Continued) | | | | |
| --- | --- | --- | --- | --- |
| Gene Replaced (Gene number) | Parent Strains | | | |
|  |  |  |  |  |
| YDL074C  (3771) | BY4743 | K8409 | EUF153-RAD5^+^ | EUF180-RAD5^+^ |
|  | SK1 | K4387 | FK192 | FK193 |
|  |  |  |  |  |
| YDL156W  (3854) | BY4743 | K8409 | EUF153-RAD5^+^ | EUF180-RAD5^+^ |
|  |  |  |  |  |
| YDL213C  (3911) | BY4743 | K8409 | EUF153-RAD5^+^ | EUF180-RAD5^+^ |
|  |  |  |  |  |
| YDR014W  (3953) | BY4743 | K8409 | EUF153-RAD5^+^ | EUF180-RAD5^+^ |
|  |  |  |  |  |
| YDR022C  (3961) | BY4743 | K8409 | EUF153-RAD5^+^ | EUF180-RAD5^+^ |
|  |  |  |  |  |
| YDR143C  (4077) | BY4743 | K8409 | EUF153-RAD5^+^ | EUF180-RAD5^+^ |
|  |  |  |  |  |
| YDR206W  (3565) | BY4743 | K8409 | EUF153-RAD5^+^ | EUF180-RAD5^+^ |
|  |  |  |  |  |
| YDR279W  (3638) | BY4743 | K8409 | EUF153-RAD5^+^ | EUF180-RAD5^+^ |
|  |  |  |  |  |
| YDR289C  (3648) | BY4743 | K8409 | EUF153-RAD5^+^ | EUF180-RAD5^+^ |
|  |  |  |  |  |
| YDR359C  (4196) | BY4743 | K8409 | EUF153-RAD5^+^ | EUF180-RAD5^+^ |
|  | SK1 | K4387 | FK192 | FK193 |
|  |  |  |  |  |
| YEL023C  (0264) | BY4743 | K8409 | EUF153-RAD5^+^ | EUF180-RAD5^+^ |
|  |  |  |  |  |
| YER041W  (0174) | BY4743 | K8409 | EUF153-RAD5^+^ | EUF180-RAD5^+^ |
|  |  |  |  |  |
| YER088C  (6393) | BY4743 | K8409 | EUF153-RAD5^+^ | EUF180-RAD5^+^ |

| Table S4.1 *Saccharomyces cerevisiae* *KANMX4* Deletion Strains (Continued) | | | | |
| --- | --- | --- | --- | --- |
| Gene Replaced (Gene number) | Parent Strains | | | |
|  |  |  |  |  |
| YER092W  (6397) | BY4743 | K8409 | EUF153-RAD5^+^ | EUF180-RAD5^+^ |
|  |  |  |  |  |
| YFL013C  (7279) | BY4743 | K8409 | EUF153-RAD5^+^ | EUF180-RAD5^+^ |
|  |  |  |  |  |
| YGL066W  (4433) | BY4743 | K8409 | EUF153-RAD5^+^ | EUF180-RAD5^+^ |
|  | SK1 | K4387 | FK192 | FK193 |
|  |  |  |  |  |
| YGL127C  (4494) | BY4743 | K8409 | EUF153-RAD5^+^ | EUF180-RAD5^+^ |
|  | FK3035 | FK3036 |  |  |
|  |  |  |  |  |
| YGL167C (1 – 2,233 bp)  (167S) | BY4743 | K8409 | EUF153-RAD5^+^ | EUF180-RAD5^+^ |
|  |  |  |  |  |
| YGL167C (overlaps with 152 – 333 bp of YGL168W)  (4534) | K8409 | EUF153-RAD5^+^ | EUF180-RAD5^+^ |  |
|  |  |  |  |  |
| YGL168W (1 – 81 bp)  (168S) | BY4743 | K8409 | EUF153-RAD5^+^ | EUF180-RAD5^+^ |
|  | SK1 |  |  |  |
|  |  |  |  |  |
| YGL168W (overlaps with 2,673 – 2,853 bp of YGL167C)  (4535) | K8409 | EUF153-RAD5^+^ | EUF180-RAD5^+^ |  |
|  |  |  |  |  |
| YGL250W  (4617) | BY4743 | K8409 | EUF153-RAD5^+^ | EUF180-RAD5^+^ |
|  | FK3035 | FK3036 |  |  |
|  |  |  |  |  |
| YGR067C  (4697) | BY4743 | K8409 | EUF153-RAD5^+^ | EUF180-RAD5^+^ |
| Table S4.1 *Saccharomyces cerevisiae* *KANMX4* Deletion Strains (Continued) | | | | |
| Gene Replaced (Gene number) | Parent Strains | | | |
|  |  |  |  |  |
| YGR068C  (4698) | BY4743 | K8409 | EUF153-RAD5^+^ | EUF180-RAD5^+^ |
|  |  |  |  |  |
| YGR071C  (4701) | BY4743 | K8409 | EUF153-RAD5^+^ | EUF180-RAD5^+^ |
|  |  |  |  |  |
| YGR134W  (6405) | BY4743 | K8409 | EUF153-RAD5^+^ | EUF180-RAD5^+^ |
|  |  |  |  |  |
| YHL023C  (0940) | BY4743 | K8409 | EUF153-RAD5^+^ | EUF180-RAD5^+^ |
|  | SK1 | K4387 | FK192 | FK193 |
|  |  |  |  |  |
| YHR033W  (0996) | BY4743 | K8409 | EUF153-RAD5^+^ | EUF180-RAD5^+^ |
|  |  |  |  |  |
| YHR100C  (1928) | BY4743 | K8409 | EUF153-RAD5^+^ | EUF180-RAD5^+^ |
|  |  |  |  |  |
| YIL007C  (1399) | BY4743 | K8409 | EUF153-RAD5^+^ | EUF180-RAD5^+^ |
|  |  |  |  |  |
| YIL152W  (2311) | BY4743 | K8409 | EUF153-RAD5^+^ | EUF180-RAD5^+^ |
|  |  |  |  |  |
| YJL047C  (1376) | BY4743 | K8409 | EUF153-RAD5^+^ | EUF180-RAD5^+^ |
|  |  |  |  |  |
| YKL054C  (4903) | BY4743 | K8409 | EUF153-RAD5^+^ | EUF180-RAD5^+^ |
|  | SK1 | K4387 | FK192 | FK193 |
|  | FK3035 | FK3036 |  |  |
|  |  |  |  |  |
| YKL056C  (4905) | BY4743 | K8409 | EUF153-RAD5^+^ | EUF180-RAD5^+^ |
|  |  |  |  |  |
| YKL098W  (4948) | BY4743 | K8409 | EUF153-RAD5^+^ | EUF180-RAD5^+^ |
|  |  |  |  |  |
| YKR051W  (5122) | BY4743 | K8409 | EUF153-RAD5^+^ | EUF180-RAD5^+^ |

| Table S4.1 *Saccharomyces cerevisiae* *KANMX4* Deletion Strains (Continued) | | | | |
| --- | --- | --- | --- | --- |
| Gene Replaced (Gene number) | Parent Strains | | | |
|  |  |  |  |  |
| YKR077W  (5993) | BY4743 | K8409 | EUF153-RAD5^+^ | EUF180-RAD5^+^ |
|  |  |  |  |  |
| YLL049W  (6426) | BY4743 | K8409 | EUF153-RAD5^+^ | EUF180-RAD5^+^ |
|  |  |  |  |  |
| YLR003C  (1558) | BY4743 | K8409 | EUF153-RAD5^+^ | EUF180-RAD5^+^ |
|  |  |  |  |  |
| YLR016C  (1571) | BY4743 | K8409 | EUF153-RAD5^+^ | EUF180-RAD5^+^ |
|  |  |  |  |  |
| YLR052W  (6441) | BY4743 | K8409 | EUF153-RAD5^+^ | EUF180-RAD5^+^ |
|  |  |  |  |  |
| YLR097C  (2708) | BY4743 | K8409 | EUF153-RAD5^+^ | EUF180-RAD5^+^ |
|  |  |  |  |  |
| YLR125W  (2736) | BY4743 | K8409 | EUF153-RAD5^+^ | EUF180-RAD5^+^ |
|  |  |  |  |  |
| YLR224W  (4173) | BY4743 | K8409 | EUF153-RAD5^+^ | EUF180-RAD5^+^ |
|  |  |  |  |  |
| YLR247C  (5156) | BY4743 | K8409 | EUF153-RAD5^+^ | EUF180-RAD5^+^ |
|  |  |  |  |  |
| YLR254C  (5163) | BY4743 | K8409 | EUF153-RAD5^+^ | EUF180-RAD5^+^ |
|  |  |  |  |  |
| YLR315W  (5224) | BY4743 | K8409 | EUF153-RAD5^+^ | EUF180-RAD5^+^ |
|  |  |  |  |  |
| YLR320W  (5229) | BY4743 | K8409 | EUF153-RAD5^+^ | EUF180-RAD5^+^ |
|  |  |  |  |  |
| YLR376C  (5285) | BY4743 | K8409 | EUF153-RAD5^+^ | EUF180-RAD5^+^ |
|  |  |  |  |  |
| YLR392C  (5301) | BY4743 | K8409 | EUF153-RAD5^+^ | EUF180-RAD5^+^ |
|  |  |  |  |  |
| YLR413W  (5321) | BY4743 | K8409 | EUF153-RAD5^+^ | EUF180-RAD5^+^ |
| Table S4.1 *Saccharomyces cerevisiae* *KANMX4* Deletion Strains (Continued) | | | | |
| Gene Replaced (Gene number) | Parent Strains | | | |
|  |  |  |  |  |
| YLR437C  (6040) | BY4743 | K8409 | EUF153-RAD5^+^ | EUF180-RAD5^+^ |
|  |  |  |  |  |
| YMR147W  (0728) | BY4743 | K8409 | EUF153-RAD5^+^ | EUF180-RAD5^+^ |
|  |  |  |  |  |
| YMR179W  (0764) | BY4743 | K8409 | EUF153-RAD5^+^ | EUF180-RAD5^+^ |
|  |  |  |  |  |
| YMR206W  (0792) | BY4743 | K8409 | EUF153-RAD5^+^ | EUF180-RAD5^+^ |
|  |  |  |  |  |
| YMR233W  (0819) | BY4743 | K8409 | EUF153-RAD5^+^ | EUF180-RAD5^+^ |
|  |  |  |  |  |
| YNL094W  (2982) | BY4743 | K8409 | EUF153-RAD5^+^ | EUF180-RAD5^+^ |
|  |  |  |  |  |
| YNL133C  (2943) | BY4743 | K8409 | EUF153-RAD5^+^ | EUF180-RAD5^+^ |
|  |  |  |  |  |
| YNL157W  (2055) | BY4743 | K8409 | EUF153-RAD5^+^ | EUF180-RAD5^+^ |
|  |  |  |  |  |
| YNL215W  (1997) | BY4743 | K8409 | EUF153-RAD5^+^ | EUF180-RAD5^+^ |
|  |  |  |  |  |
| YNL288W  (1156) | BY4743 | K8409 | EUF153-RAD5^+^ | EUF180-RAD5^+^ |
|  |  |  |  |  |
| YNR051C  (6078) | BY4743 | K8409 | EUF153-RAD5^+^ | EUF180-RAD5^+^ |
|  |  |  |  |  |
| YOR220W  (2476) | BY4743 | K8409 | EUF153-RAD5^+^ | EUF180-RAD5^+^ |
|  |  |  |  |  |
| YPL017C  (7339) | BY4743 | K8409 | EUF153-RAD5^+^ | EUF180-RAD5^+^ |
|  |  |  |  |  |
| YPL055C  (2773) | BY4743 | K8409 | EUF153-RAD5^+^ | EUF180-RAD5^+^ |
|  | SK1 | K4387 | FK192 | FK193 |
|  |  |  |  |  |
| YPL095C  (2157) | BY4743 | K8409 | EUF153-RAD5^+^ | EUF180-RAD5^+^ |
| Table S4.1 *Saccharomyces cerevisiae* *KANMX4* Deletion Strains (Continued) | | | | |
| Gene Replaced (Gene number) | Parent Strains | | | |
|  |  |  |  |  |
| YPR045C  (5464) | BY4743 | K8409 | EUF153-RAD5^+^ | EUF180-RAD5^+^ |
|  |  |  |  |  |
| YPR152C  (5567) | BY4743 | K8409 | EUF153-RAD5^+^ | EUF180-RAD5^+^ |
|  |  |  |  |  |
| YPR179C  (5594) | BY4743 | K8409 | EUF153-RAD5^+^ | EUF180-RAD5^+^ |
|  |  |  |  |  |
| YGL249W (ZIP2) CONTROL  (4616) | BY4743 | K8409 | EUF153-RAD5^+^ | EUF180-RAD5^+^ |
|  |  |  |  |  |
| YIL132C (CSM2) CONTROL  (2291) | BY4743 | K8409 | EUF153-RAD5^+^ | EUF180-RAD5^+^ |
|  |  |  |  |  |
| YLR329W (REC104) CONTROL  (5238) | BY4743 | K8409 | EUF153-RAD5^+^ | EUF180-RAD5^+^ |
|  |  |  |  |  |
| YMR048W (CSM3) CONTROL  (6740) | BY4743 | K8409 | EUF153-RAD5^+^ | EUF180-RAD5^+^ |
|  |  |  |  |  |
| YMR133W (REC114) CONTROL  (6574) | BY4743 | K8409 | EUF153-RAD5^+^ | EUF180-RAD5^+^ |
|  |  |  |  |  |
| YPL200W (CSM4) CONTROL  (1092) | BY4743 | K8409 | EUF153-RAD5^+^ | EUF180-RAD5^+^ |

**Strain Nomenclature**

All strains are given the parent strain accession (e.g. E0) followed by a unique four digit number corresponding to the ORF that is replaced with the *KANMX4* cassette (e.g. 1092, making this example strain name E01092).
